# Anthracenedione Derivatives as Anticancer Agents Isolated from Secondary Metabolites of the Mangrove Endophytic Fungi

**DOI:** 10.3390/md8041469

**Published:** 2010-04-23

**Authors:** Jian-ye Zhang, Li-yang Tao, Yong-ju Liang, Li-ming Chen, Yan-jun Mi, Li-sheng Zheng, Fang Wang, Zhi-gang She, Yong-cheng Lin, Kenneth Kin Wah To, Li-wu Fu

**Affiliations:** 1 State Key Laboratory for Oncology in South China, Cancer Center, Sun Yat-Sen University, Guangzhou, 510060, China; E-Mails: jianyez2003@yahoo.com.cn (J.Z.); sohutly@163.com (L.T.); liangyju@mail.sysu.edu.cn (Y.L.); chlm78@yahoo.com.cn (L.C.); myjgj_77@163.com (Y.M.); lesley860221@163.com (L.Z.); wangfang0203@163.com (F.W.); 2 School of Chemistry and Chemical Engineering, Sun Yat-Sen University, Guangzhou, 510275, China; 3 School of Pharmacy, The Chinese University of Hong Kong, Hong Kong, China; E-Mail: kennethto@cuhk.edu.hk (K.K.W.T.)

**Keywords:** mangrove endophytic fungi, anthracenedione derivatives, anticancer, apoptosis, structure-activity relationship

## Abstract

In this article, we report anticancer activity of 14 anthracenedione derivatives separated from the secondary metabolites of the mangrove endophytic fungi *Halorosellinia* sp. (No. 1403) and *Guignardia* sp. (No. 4382). Some of them inhibited potently the growth of KB and KBv200 cells, among which compound **6** displayed strong cytotoxicity with IC_50_ values of 3.17 and 3.21 μM to KB and KBv200 cells, respectively. Furthermore, we demonstrate that the mechanism involved in the apoptosis induced by compound 6 is probably related to mitochondrial dysfunction. Additionally, the structure-activity relationships of these compounds are discussed.

## 1. Introduction

Cancer has become an increasing public health problem due to its high rates of morbidity and mortality. Conventional cancer chemotherapy has the limitation of multidrug resistance (MDR) caused by overexpression of integral membrane transporters, such as P-gp, which can efflux intracellular anticancer drugs thus decreasing drug accumulation. MDR cells are resistant to cytotoxic effects of various structurally and mechanistically unrelated chemotherapeutic agents [1–3]. Developing new anticancer drugs that are efficient to MDR cells is a feasible strategy to overcome MDR.

The natural environment is still the most important supply of novel drugs despite development of combinatorial chemistry, which can quickly generate thousands of new chemicals. The terrestrial environment has been mined for drugs for many years with great success. Now humans have recognized that the oceans are a rich source of natural products with potential as drugs [4–6]. Many promising compounds of new and complicated structure types have been isolated from the oceans and some have been identified as leading preclinical anticancer compounds. Interestingly, marine microalgae, cyanobacteria, and heterotrophic bacteria living in association with invertebrates (e.g., sponges, tunicates, and soft corals) have been identified, or strongly suspected, as the true sources of many bioactive and useful constituents [7,8]. Cell sorting, culture, and molecular biological methods are helpful to clarify the intriguing aspect of marine metabolism [9–11]. Marine-derived fungi have been rich sources of structurally novel and biologically active secondary metabolites, which have become attractive as important resources for new chemicals in drug discovery [12,13]. As part of our ongoing investigations directed toward the discovery of structurally new and biologically active natural products from marine endophytic fungi, we studied the anthracenedione derivatives acting as the potent anticancer agents screened from the mangrove endophytic fungus *Halorosellinia* sp. (No. 1403) and *Guignardia* sp. (No. 4382). We have reported the isolation and identification of these compounds before [13–15]. Here, the mechanisms involving the apoptosis induced by these anthracenedione derivatives are investigated and discussed. Moreover, we analyze the structure-activity relationships (SAR) of these compounds.

## 2. Results and Discussion

**Figure 1:**
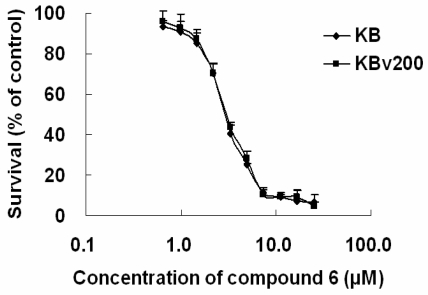
Compound 6 showed potent cytotoxicity to KB and KBv200 cells. Cytotoxicity was measured by 3-(4,5-Dimethyl-2-thiazolyl)2,5-diphenyl-2H-tetrazolium bromide (MTT) assay. The cells having grown for 24 h were exposed to a full range of concentrations of compound 6 for 68 h. After subsequent staining with MTT for 4 h, cell viability was assessed by Model 550 Microplate reader Results were shown as means ± SD of at least triplicate determinations. Each experiment was performed in three replicate wells.

In this study, we investigated the anticancer activity of several anthracenedione derivatives, which was separated from the secondary metabolites of the mangrove endophytic fungus *Halorosellinia* sp. (No. 1403) and *Guignardia* sp. (No. 4382). Compounds 1–14 of 9,10-anthracenediones showed potent cytotoxicity to drug-sensitive parental KB cells and MDR KBv200 cells (Table 1), among which compound 6 displayed strong cytotoxicity with the IC_50_ values of 3.17 and 3.21 μM to KB and KBv200 cells, respectively. Anthraquinones represent a large family of compounds having diverse biological activities [16–18]. Herein, compound 6 displayed its cytotoxicity to KB and KBv200 cells (Figure 1).

Our results indicate that various substituting groups made their different contributions to the anticancer activity. It is believed that hydroxy groups perform important roles by offering hydrogen bonding with biomacromolecules such as proteins. At the same time, the quantity and location of hydroxy groups are fundamental to the activities of the compounds [19]. Compound 1, 5, 6, 7 and 9 potently inhibited growth of KB cells and KBv200 cells. Compound 6, containing 1-hydroxy, showed the most potent inhibition of growth of KB cells and KBv200 cells. Interestingly, compounds with 1-hydroxy and another hydroxy at other carbons led to the decrease of anticancer activity, such as compound 1, 3, 7 and 9. Compounds 4 and 10, with multiple substituting groups at different carbons, showed its cytotoxicity IC_50_ values more than 500.0 μM. Compared to compounds 4 and 10 (containing three hydroxy groups), compound 9 has two hydroxy groups, which is probably related to the differences observed in IC_50_ values. It seems that multi-hydroxy-substitution was unfavorable to the cytotoxicity activity. Compound 6 exhibited similar cytotoxicity to both the drug-sensitive parental KB cells and the MDR KBv200 cells. Compared to KB cells, KBv200 cells showed resistance to compounds 1, 3, 7 and 14.

**Figure 2:**
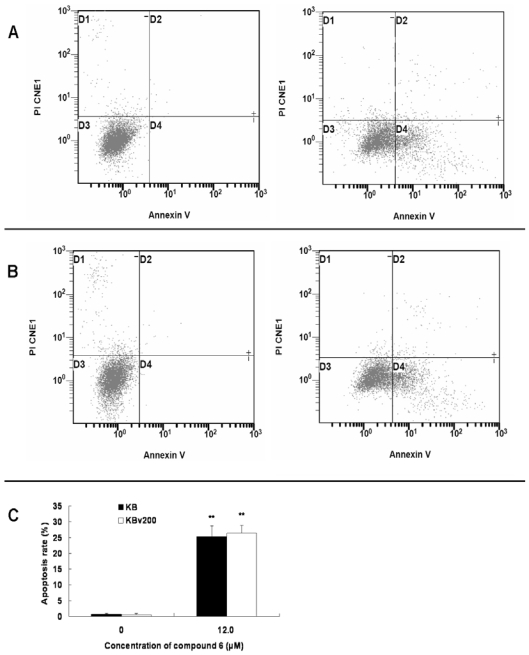
Compound 6 induced cell apoptosis in KB and KBv200 cells. Compound 6 induced cell apoptosis in KB and KBv200 cells detected by Annexin V-FITC/PI double staining and flow cytometer assay. The right bottom quadrant represented cells stained mainly by Annexin-V (early apoptotic cells) and the top right quadrant represented cells stained by both PI and Annexin-V (late apoptotic/necrotic secondary necrosis). The top left quadrant represented cells stained mainly by PI and viable cells negative for both Annexin-V and PI appeared in the left bottom quadrant. (**A**) KB cells exposed to 0 and 12 μM compound 6, respectively. (**B**) of KBv200 cells exposed to 0 and12 μM compound 6, respectively. (**C**) statistical analysis of the above results. The apoptosis rate is showed in the bar graph. The results represent the data of three assays (means ± SD). * *P* < 0.05 and **P < 0.01 *versus* the control.

**Figure 3:**
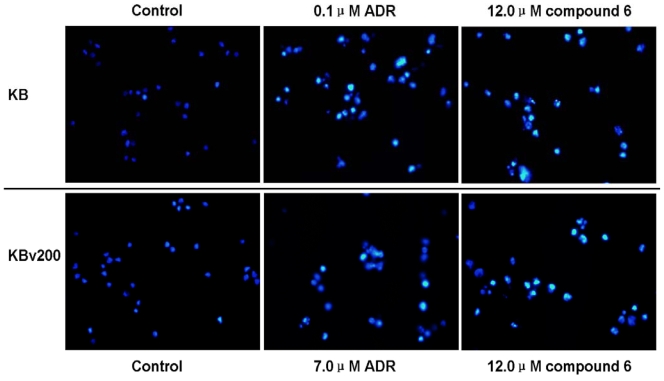
Compound 6 induces morphological changes representative of apoptosis. Cell apoptosis induced by compound 6 was examined by Hoechst 33258 staining and observed under fluorescence microscope at 400 × magnification. KB cells and KBv200 cells were treated with compound 6 of 12.0 μM for 48 h. The apoptotic cells detected by the fluorescence microscopy displayed typical changes of apoptosis including staining bright of condensed or fragmented nucleus. Herein, treatment with adriamycin (0.1 μM for KB cells and 7.0 μM for KBv200 cells) for 48 h was included as the positive control.

In addition to the SAR, the mechanisms involved in the anticancer activity of compounds 1–14 is attractive to investigate. Anthraquinones exhibit anticancer activity through various pathways including inducing apotosis [18]. Indeed, compound 6 induced apoptosis in KB and KBv200 cells (Figure 2). After KB and KBv200 cells were treated with 12.0 μM compound 6 for 48 h, the cells were gathered and exposed to double staining and flow cytometry analysis. The results showed that apoptosis rates of drug-treated cells were 25.3 ± 3.5% and 26.4 ± 2.4% for KB and KBv200 cells, respectively. Thus, compared to the control experiments, compound 6 significantly increased the apoptosis rate in KB and KBv200 cells. To confirm the apoptosis-induction of compound 6, we carried out Hoechst 33258 staining assay and observed the cells by fluorescence microscopy. Control cells showed an even distribution of staining of homogeneous nuclei features. Apoptotic cells displayed typical changes of apoptosis including staining bright of condensed or fragmented nucleus (Figure 3).

**Figure 4:**
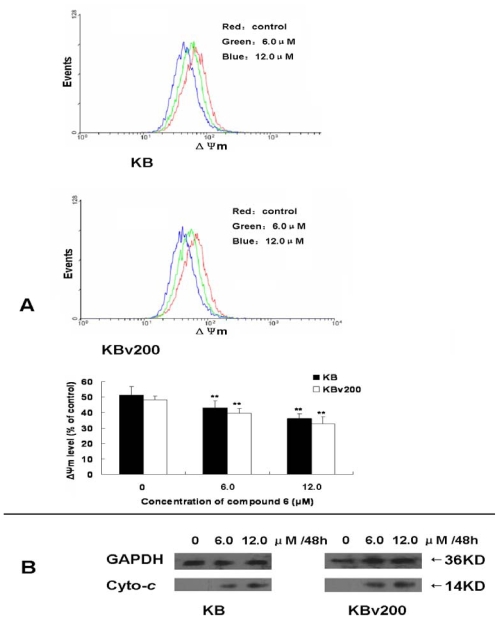
Compound 6 treatment results in the loss of ΔΨ_m_ and release of cytochrome *c*. After cells were exposed to 0, 6.0 and 12.0 μM of compound 6 for 48 h, the ΔΨ_m_ was determined by flow cytometry. Loss of ΔΨ_m_ in KB and KBv200 cells and release of cytochrome *c* were observed in a dose-dependent manner. (**A**) the decrease of ΔΨ_m_ in KB and KBv200 cells; also, ΔΨ_m_ levels expressed as units of MFI were calculated as percentage of control. Results are mean ± SD of three determinations. * *P* < 0.05 and ** *P* < 0.01 *vs.* the control. (**B**) the release of cytochrosome *c* after treatment with compound 6 for the indicated time in KB cells and KBv200 cells, respectively. GAPDH detection was used to confirm equal protein loading.

Chemotherapeutic agents can usually induce apoptosis *via* a mitochondrial pathway [20]. In this article, compound 6 treatment led to loss of mitochondrial potential (ΔΨ_m_). Herein, DiOC6 was performed as the mitochondria-specific and voltage-dependent dye for determining ΔΨ_m_, the loss of which is regarded as a leading important factor in the apoptotic pathway. After KB and KBv200 cells were treated with compound 6 at the concentrations of 0, 6.0 and 12.0 μM for 48 h, the decrease of ΔΨ_m_ showing was observed in a concentration-dependent manner (Figure 4A). The levels of ΔΨ_m_ were 83.91 ± 9.18%, 70.50 ± 5.99% of control in KB cells, 81.59 ± 6.23%, 67.49 ± 9.49% of control in KBv200 cells, demonstrating that compound 6 caused mitochondrial dysfunction in KB and KBv200 cells. Mitochondrial dysfunctions, including loss of ΔΨ_m_, are likely to cause apoptosis [21,22]. Under current understanding, loss of ΔΨ_m_ causes release of cytochrosome *c* and sequent activation of caspases [23]. Indeed, release of cytochrosome *c* was observed after exposure to compound 6 at the indicated concentrations for 48 h in a concentration-dependent manner (Figure 4B). Taken together, compound 6 might induce apoptosis *via* a mitochondrial pathway.

**Figure 5:**
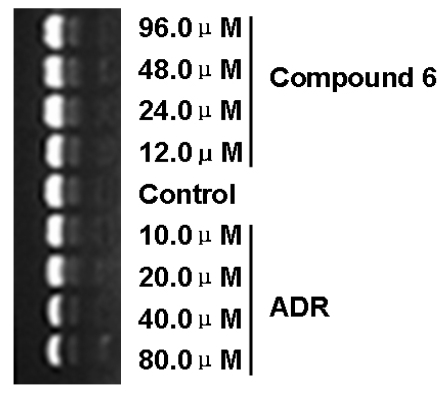
Compound 6 does not intercalate into DNA. After 10.0, 20.0, 40.0, 80.0 μM ADR and 12.0, 24.0, 48.0, 96.0 μM compound 6 at 37 °C for 48 h, the mixture was subjected to electrophoresis on a 1.2% agarose gel stained with 1.5 μM EB at 60 V for 40 min in 1 × TAE buffer and photographed under ultraviolet light.

As important cancer chemotherapy drugs, anthracyclines have a wide spectrum of anticancer activity, and to date have already played an important role in cancer treatment [24–26]. It is well known that anthracyclines, such as adriamycin (ADR) and daunomycin (DAM) can intercalate into DNA. There is compelling evidence that cellular DNA is the primary target of these drugs [27,28]. To clarify whether compound 6 exerted cytotoxicity to KB cells and KBv200 cell *via* targeting DNA, a DNA binding assay was performed. The results indicated that compound 6 did not intercalate into DNA and implied that apoptosis induced by compound 6 might not involve DNA intercalation (Figure 5).

Anthracenedione derivatives have been extensively investigated for various activities and different mechanisms, such as inducing apoptosis, modulating G-quadruplex recognition, and inhibiting endothelial cell proliferation [29,30]. In this article, we focused on the cytotoxicity of anthracenedione derivatives compound 1–14, discussion of SAR and mechanism of inducing apoptosis. The mechanism involving the apoptosis induced by compound 6 is probably related to mitochondrial dysfunction.

## 3. Experimental Section

### 3.1. Chemicals and reagents

3-(4,5-Dimethyl-2-thiazolyl)2,5-diphenyl-2H-tetrazolium bromide (MTT) was purchased from Sigma Chemical Co. Compounds for MTT assays were obtained with a purity of >98% from the secondary metabolites of the mangrove endophytic fungus *Halorosellinia* sp. (No. 1403) and *Guignardia* sp. (No. 4382). KB and KBv200 are human epidermoid carcinoma cell lines. KBv200 cells, the classic multidrug resistant cell line expressing high levels of P-gp, were cloned from drug-sensitive parental KB cells by stepwise exposure to increasing doses of vincristine (VCR) and ethylmethane sulfonate (EMS) mutagenesis. Compared with the KB cell line, the KBv200 cell line was about 100-times more resistant to VCR. KB cells and KBv200 cells, obtained from the Chinese Academy of Medical Sciences (Beijing, China), were cultured in RPMI 1640 medium containing 100 U/mL penicillin, 100 μg/mL streptomycin, and 10% fetal bovine serum (FBS). All cells were maintained in a humidified atmosphere incubator containing 5% CO_2_ and 95% air at 37 °C [33].

### 3.2. Fermentation of fungi, extraction, isolation and identification of compounds

Starter cultures (from Prof. L.L.P. Vrimjoed) were maintained on cornmeal seawater agar. Plugs of agar supporting mycelial growth were cut and transferred aseptically to a 250 mL Erlenmeyer flask containing 100 mL of liquidmedium (glucose 10 g/L, peptone 2 g/L, yeast extract 1 g/L, NaCl 2 g/L, pH 7.0). The flask was incubated at 28 °C. After shaking on a rotary shaker for 3–5 days, the mycelium was aseptically transferred to a 500 mL Erlenmeyer flask containing the culture liquid (200 mL). The flask was then incubated at 28 °C for 25 days. The cultures (150 L) were filtered through cheesecloth. The filtrate was concentrated to 5 L below 50 °C and extracted three times by shaking with an equal volume of ethyl acetate. The combined organic extracts were subjected to a silica gel column, eluting with a gradient of petroleum ether to ethyl acetate [13,14].

NMR data were recorded on a Varian Inova-500 NB spectrometer, using DMSO or CDCl_3_ as solvent and TMS as internal standard. Mass spectra were acquired on a VG-ZAB mass spectrometer. IR spectra were obtained on a Nicolet 5DX-FTIR spectrophotometer [14].

### 3.3. Cell viability assay

Cells were harvested during logarithmic growth phase and seeded in 96-well plates at a density of 1.5 × 10^4^ cells/mL in a final volume of 190 μL/well. After 24 h incubation, 10 μL of tested compounds covering a full range of concentrations were added to 96-well plates. After 68 h treatment, 10 μL MTT (10 mM stock solution of saline) was added to each well for 4 h. Subsequently, the supernatant was removed, and MTT crystals were dissolved with 100 μL anhydrous DMSO in each well. Thereafter, cell viability was measured using a Model 550 Microplate reader (BIO-RAD, USA) at 540 nm with 655 nm as reference filter. Experiments were performed at least three times. The 50% inhibitory concentration (IC_50_) was determined as the anticancer drug concentration causing 50% reduction of cell viability and calculated from the cytotoxicity curves (Bliss’s software) [32]. Cell survival was calculated using the following formula: survival (%) = (mean experimental absorbance/mean control absorbance) × 100%.

### 3.4. Determination of mitochondrial potential (ΔΨ_m_)

ΔΨ_m_ was measured by flow cytometry with the mitochondrial tracking fluorescent DiOC6. The cationic lipophilic fluorochrome DiOC6 is a cell permeable marker that specifically accumulates into mitochondria depending on ΔΨ_m_. After exposure to tested compounds for the indicated time, 5 × 10^5^ cells were harvested, centrifuged at 1,000 rpm for 5 min, and washed once with ice-cold PBS. Thereafter, cells were incubated with 40 nM DiOC6 at 37 °C for 20 min in the dark. Then cells were washed twice, resuspended in 1 mL PBS, and analyzed on a FACS Caliber flow cytometer (Beckman-coulter, Elite) with the excitation wavelength of 484 nm and emission wavelength of 501 nm. At least 10,000 cells were determined for each sample. The data obtained from flow cytometry were analyzed by CellQuest software and expressed as mean fluorescence intensity (MFI) [31]. The expressed data were the results of at least three independent determinations.

### 3.5. Annexin V-FITC/PI assay

Apoptosis rate was quantified by detecting surface exposure of phosphatidylserine in apoptotic cells using Annexin V-FITC/PI (propidium iodide) apoptosis detection kit (BD Biosciences Clontech) according to the manufacturer’s instruction. After KB and KBv200 cells were treated with 12 μM compound 6 for 48 h, the cells were collected and washed twice with cold PBS. Then, 5 × 10^5^ cells were resuspended in 0.5 mL binding buffer containing Annexin-V (1:50 according to the manufacturer’s instruction) and 40 ng/sample of PI for 20 min at 37 °C in the dark. The numbers of viable, apoptotic and necrotic cells were quantified by flow cytometry (Beckman Coulter, Cytomics FC500, USA) and analysis by the CellQuest software. At least 10,000 cells were calculated for each sample. The apoptosis rate (%) = (the number of apoptotic cells/the number of total cells observed) × 100% [18].

### 3.6. Hoechst 33258 staining

After treatment with or without compound 6, both floating and trypsinized adherent cells were collected, washed once with ice-cold PBS, fixed with 1 mL 4% paraformaldehyde for 20 min, then washed once with ice-cold PBS. After that, the cells were incubated in 1 mL PBS containing 10 μM Hoechst 33258 at 37 °C for 30 min, washed twice, and observed using fluorescence microscopy with standard excitation filters (Leica Dmirb, Germany) in random microscopic fields at 400 × magnification. The experiments were performed at least three times [28].

### 3.7. Subcellular fractionation for western blot analysis of cytosolic cytochrome c

Cells were treated with tested compounds for the indicated time and harvested by centrifugation at 1,000 rpm for 5 min. The pellets were washed twice with ice-cold PBS, suspended with 5-fold volume of ice-cold cell extract buffer (20 mM Hepes–KOH (pH 7.5), 10 mM KCl, 1.5 mM MgCl2, 1 mM EDTA, 1 mM EGTA, 1 mM DTT, 250 mM sucrose, 0.1 mM PMSF and 0.02 mM aprotinin) and incubated for 40 min at 4 °C. Then the cells were centrifuged at 1,200 rpm for 10 min at 4 °C; the supernatant was subsequently centrifuged at 12,000 rpm for 15 min at 4 °C and the final supernatant was used as cytosolic fraction. Then 5 × loading buffer (250 mM Tris-Cl (pH 6.8), 50% glycerol, 10% sodium dodecylsulphate, 1.25‰ bromphenol blue, 0.5 M dithiothreitol) was added to the above supernatant and the mixture was boiled at 100 °C for 15 min. After that, the protein solution was used for identification of cytosolic cytochrome c by immunoblotting with 10% SDS–PAGE and blotting onto PVDF membrane. The cytochrome c protein was detected by using anti-cytochrome c antibody (Cell Signalling, USA) in the ratio of 1:1,000 [28].

### 3.8. DNA binding assay

ADR, which is able to intercalate into nucleic bases, was used as the positive control in the DNA binding assay. Briefly, 10.0, 20.0, 40.0 and 80.0 μM ADR or 12.0, 24.0, 48.0, 96.0 μM compound 6 were incubated with pEGFPC2-H1-MDR1shDNA at 37 °C for 48 h, respectively. Then, the mixture was subjected to electrophoresis on a 1.2% agarose gel stained with 1.5 μM EB at 60 V for 40 min with 1 × TAE buffer. Observation and photography were developed with the gel imaging and analysis system (Vilber Lourmat, France) [28].

## 4. Conclusions

In summary, we screened some potent antitumor agents of 9,10-anthracenediones from secondary metabolites of the mangrove endophytic fungus. The information stemming from the structure–activity relationships will constitute the basis for the design of future compounds with potentially better activity. Our mechanistic investigation indicates that compound 6 might induce apoptosis *via* a mitochondrial pathway.

## Figures and Tables

**Table 1:** Structures and cytotoxicity IC_50_ values of compound 1–14.

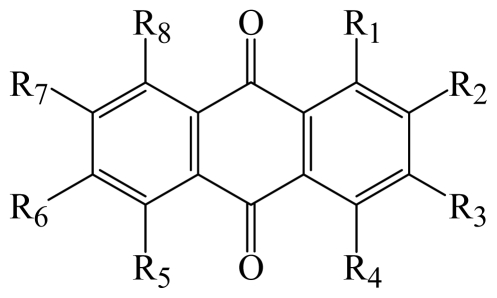									
Compound	R_1_	R_2_	R_3_	R_4_	R_6_	R_7_	R_8_	Cytotoxicity KB	IC_50_ ( μM ) KBv200
**1**	methoxy	—	methoxy	—	methyl	—	—	57.32	90.86
**2**	hydroxy	methoxy	—	hydroxy	—	methyl	—	305.14	*
**3**	hydroxy	—	methyl	—	—	—	hydroxy	174.87	331.97
**4**	hydroxy	methoxy	—	hydroxy	methyl	hydroxy	—	*	*
**5**	hydroxy	—		hydroxy	—	methyl	—	114.09	86.45
**6**	hydroxy	—	methyl	—	—	—	—	3.17	3.21
**7**	hydroxy	—	—	—	—	—	hydroxy	56.56	109.15
**8**	hydroxy	—	hydroxy	—	—	—	hydroxy	*	72.60
**9**	hydroxy	—	hydroxy	—	methoxy	—	methyl	38.05	34.64
**10**	hydroxy	—	hydroxy	—	methyl	—	hydroxy	*	*
**11**	methoxy	—	methyl	—	—	—	hydroxy	*	185.68
**12**	hydroxy	—	hydroxy	—	methyl	—	methoxy	*	190.81
**13**	hydroxy	methoxy	—	methoxy	methyl	hydroxy	—	*	301.47
**14**	hydroxy	methoxy		methoxy		methyl		68.39	243.69

* The IC_50_ values were more than 500.0 μM. As the positive control, adriamycin showed cytotoxicity to KB and KBv200 cells with the IC_50_ values of 0.034 and 1.894 μM, respectively.
